# Knowledge, attitudes, and behaviors regarding cancer screening among adults in primary care

**DOI:** 10.1186/s12889-026-28493-9

**Published:** 2026-07-10

**Authors:** Melike Karabulut Özer, Yeliz Kasko Arıcı, Özgür Enginyurt

**Affiliations:** 1 Department of Family Medicine, Ordu University Education and Research Hospital, Ordu, 52200 Türkiye; 2 Department of Biostatistics, Ordu University Faculty of Medicine, Ordu, Türkiye

**Keywords:** Cancer screening, Primary care, Family medicine, Health literacy, Preventive health services

## Abstract

**Objective:**

To assess adults’ knowledge, attitudes, and behaviors about cancer screening in primary care, and to evaluate short-term changes in attitudes after brief written information.

**Methods:**

This cross-sectional study was conducted from March to June 2019 in a family medicine outpatient clinic. Adults aged 20–70 years who agreed to participate were consecutively enrolled. Data were collected using a structured face-to-face questionnaire assessing sociodemographic characteristics, cancer screening awareness, prior screening behavior, barriers, and post-brochure attitudes. Because study participation age did not necessarily correspond to screening eligibility, a post hoc binary screening-eligibility variable was created according to the study-period age/sex framework reflected in the questionnaire and source dataset. Categorical variables were analysed using Pearson’s chi-square test or Fisher’s exact test, as appropriate, and paired pre/post willingness responses were analysed using McNemar’s exact test. Exploratory multiple binary logistic regression was performed for prior screening uptake.

**Results:**

A total of 247 participants were included (mean age 43.4 ± 12.7 years; 68.4% female). Overall, 40.5% were aware of cancer screening tests and 37.0% reported having previously undergone at least one screening test. However, uptake differed markedly by screening eligibility: 89/169 (52.7%) eligible participants versus 2/77 (2.6%) non-eligible participants had ever been screened. Awareness distribution differed significantly between healthcare workers and non-healthcare participants, whereas crude prior screening uptake did not differ significantly by occupation. Willingness to undergo screening increased after the brochure, indicating a short-term positive change in intention.

**Conclusions:**

Awareness of cancer screening did not consistently translate into screening participation. Crude non-screening rates should not be interpreted without considering screening eligibility structure and sample composition. Brief written information may improve short-term willingness, but these findings should be interpreted as intention rather than confirmed behavioral change.

**Supplementary Information:**

The online version contains supplementary material available at https://doi.org/10.1186/s12889-026-28493-9.

## Introduction

Cancer continues to constitute a substantial global burden of morbidity and mortality; according to 2022 estimates, approximately 20 million new cases and 9.7 million cancer-related deaths occur annually [1]. Early diagnosis and evidence-based screening programmes are critical to reducing this burden; however, the effectiveness of early detection strategies and screening practices depends on multilevel factors, including health system capacity, healthcare-seeking behavior, and access to services [2].

Despite the availability of screening programmes, participation remains suboptimal in many settings. This underuse is influenced by limited health literacy, insufficient knowledge, perceived absence of symptoms, practical access barriers, fear of diagnosis, and missed opportunities for counselling in primary care. For this reason, family medicine settings represent a critical context for understanding both awareness and behavioral uptake of screening.

Although population-based screening programmes for breast, cervical, and colorectal cancers are implemented in many countries, participation rates frequently remain below target levels, revealing a persistent “awareness–behavior” gap. In this context, primary care—and family medicine in particular—represents a key point of contact for increasing screening participation through continuity of care, opportunistic counselling, referral, and reminder strategies [3]. Moreover, systematic reviews have demonstrated that interventions such as digital reminders and mobile health applications may improve screening participation and screening intention [4].

In Türkiye, organised population-based screening programmes are implemented through national screening services. According to national guidance, breast cancer screening is offered to women aged 40–69 years with mammography every 2 years, cervical cancer screening is offered to women aged 30–65 years with HPV-DNA testing every 5 years, and colorectal cancer screening is offered to women and men aged 50–70 years with faecal occult blood testing every 2 years and colonoscopy every 10 years [5]. 

The primary aim of this study was to evaluate adults’ knowledge, attitudes, and behaviors regarding cancer screening. The secondary aim was to examine whether brief written information was associated with a short-term change in screening-related intention immediately after the intervention.

## Materials and methods

### Study design and setting

This was a cross-sectional, descriptive, and analytical study conducted between March and June 2019 in the Family Medicine Outpatient Clinic of Ordu University Education and Research Hospital, Ordu, Türkiye. The clinic functions as a first-contact primary care setting where adult patients present for routine outpatient care.

### Participants

Adults aged 20–70 years who attended the clinic during the study period and agreed to participate were consecutively enrolled using a convenience sampling approach. The inclusion criteria were age between 20 and 70 years and willingness to participate. The exclusion criteria were age below 20 years, age above 70 years, and refusal to participate. No participant was excluded after enrolment. However, several questionnaire items had missing responses; therefore, denominator-specific available-case analysis was used where appropriate.

### Questionnaire development and data collection

Data were collected using a structured 23-item questionnaire administered through face-to-face interviews. The questionnaire was developed by the research team using Turkish literature cited in the ethics submission together with national cancer screening materials available during the study period. The questionnaire items were informed by previously published studies on cancer screening awareness and behavior and were adapted to the Turkish national screening context. Content clarity and relevance were reviewed by the research team prior to data collection [6, 7]. 

The instrument covered three domains: sociodemographic and health-related characteristics, baseline knowledge/attitudes/behaviors regarding cancer screening, and post-brochure attitudes and willingness. The questionnaire did not undergo formal psychometric validation, and this is acknowledged as a limitation.

### Measures

The primary behavioral outcome was prior cancer screening uptake, defined as self-reported history of having undergone at least one cancer screening test. Because eligibility for study participation was broader than eligibility for specific cancer screening tests, a post hoc binary screening-eligibility variable was created according to the age/sex framework reflected in the questionnaire and coded source dataset used during the study period. Screening eligibility was defined according to national guidelines as follows: women aged 40–69 years for breast cancer screening, women aged 30–65 years for cervical cancer screening, and adults aged 50–70 years for colorectal cancer screening. Post-information responses were interpreted as short-term changes in intention rather than verified behavioral outcomes.

### Power analysis for sample size

To ensure adequate statistical power for the planned analyses, the sample size was calculated using G*Power version 3.1. As the primary analytical approach was based on chi-square tests applied to cross-tabulated categorical variables, the sample size calculation was conducted according to chi-square test assumptions. A 95% confidence level (α = 0.05), 80% statistical power (1–β = 0.80), 21 degrees of freedom, and a medium effect size (Cohen’s w = 0.30) were assumed. Under these parameters, the minimum required sample size was calculated as *n* = 238. A total of 247 participants were included in the final analysis, exceeding the minimum required sample size.

### Statistical analysis

Statistical analyses were performed using IBM SPSS Statistics for Windows, version 25 (IBM Corp., Armonk, NY, USA). Categorical variables were summarised as n (%). Pearson’s chi-square test was used to compare categorical variables, and Fisher’s exact test was applied when expected cell counts were sparse in 2 × 2 tables. For the paired pre- and post-brochure binary willingness item, McNemar’s exact test was used. Effect size for key chi-square analyses was reported as Cramer’s V. In addition, an exploratory multiple binary logistic regression analysis was performed to examine factors associated with prior screening uptake. The dependent variable was prior screening uptake (yes/no). Results are reported as adjusted odds ratios (aORs) with 95% confidence intervals (CIs). A sensitivity analysis excluding participants with a personal history of cancer was also performed. All tests were two-sided, and *p* < 0.05 was considered statistically significant.

### Ethical approval and consent to participate

This study was conducted as part of a medical specialty (residency) thesis entitled “Knowledge, Attitudes, and Behaviors of Adult Individuals Regarding Cancer Screening” (Thesis No.601881). The research protocol was approved by the Clinical Research Ethics Committee of Ordu University (decision no. 2019/45, dated March 7, 2019). Permission to conduct the study was also obtained from the relevant local health authority. Written informed consent was obtained from all participants in accordance with the principles of the Declaration of Helsinki.

## Results

A total of 247 adults were included in the study. No participant was excluded after enrolment; however, denominators varied across specific analyses because of item-level missing responses. The mean age was 43.4 ± 12.7 years (range 20–70), and 68.4% of participants were female. Most participants were married (70.0%), lived in urban areas (82.6%), and had social insurance coverage (92.3%). Nearly half reported a chronic disease requiring regular medication (47.0%), and 55.5% reported a family or close-contact history of cancer. Additional baseline characteristics are presented in Table 1.

**Table 1:** Participant Characteristics (*n* = 247)

Variable	*n* (%)
Age, mean ± SD	43.4 ± 12.7
Female	169/247 (68.4)
Married	173/247 (70.0)
University educated	96/247 (38.9)
Healthcare worker	78/247 (31.6)
Urban residence	204/247 (82.6)
Social insurance	228/247 (92.3)
Chronic disease	116/247 (47.0)
Personal cancer history	12/247 (4.9)
Family cancer history	137/247 (55.5)

Data are presented as n/N (%) unless otherwise indicated

Overall, 100/247 (40.5%) of participants were in the aware category, 91/247 (36.8%) were partly aware, and 56/247 (22.7%) were not aware of cancer screening tests. Among reported information sources, family physicians, social media, and nurses or other health personnel were the most frequently cited. Item-level distributions are summarised in Table 2.

**Table 2:** Awareness of Cancer Screening and Information Sources

Variable	*n* (%)
Aware of cancer screening	100/247 (40.5)
Partly aware	91/247 (36.8)
Not aware	56/247 (22.7)
Family physician	53/243 (21.8)
Social media	52/243 (21.4)
Nurse or health personnel	51/243 (21.0)

Awareness categories are presented for all participants (*n* = 247). Information-source items were multi-response variables. The three-level awareness distribution (aware / partly aware / not aware) differed significantly according to occupation, χ2(2, *N* = 247) = 45.69, *p* < 0.001, Cramer's V = 0.43

Awareness distribution also differed significantly according to occupation. The three-level awareness variable (aware / partly aware / not aware) showed a significant association with healthcare-worker status, χ²(2, *N* = 247) = 45.69, *p* < 0.001, Cramer’s V = 0.43. Descriptively, 55/78 (70.5%) healthcare workers and 45/169 (26.6%) non-healthcare participants were in the aware category. Beyond occupation, awareness also differed by age group, marital status, and education level, but not by sex. Likewise, the distribution of information sources varied according to age group, marital status, education level, and occupation (Table 2).

Among participants with available responses, 91/246 (37.0%) reported having previously undergone at least one cancer screening test, whereas 155/246 (63.0%) reported never having been screened. However, because the study inclusion age range was broader than the age/sex structure of routine screening eligibility, this crude proportion was reinterpreted according to a post hoc eligibility variable.

**Fig. 1:**
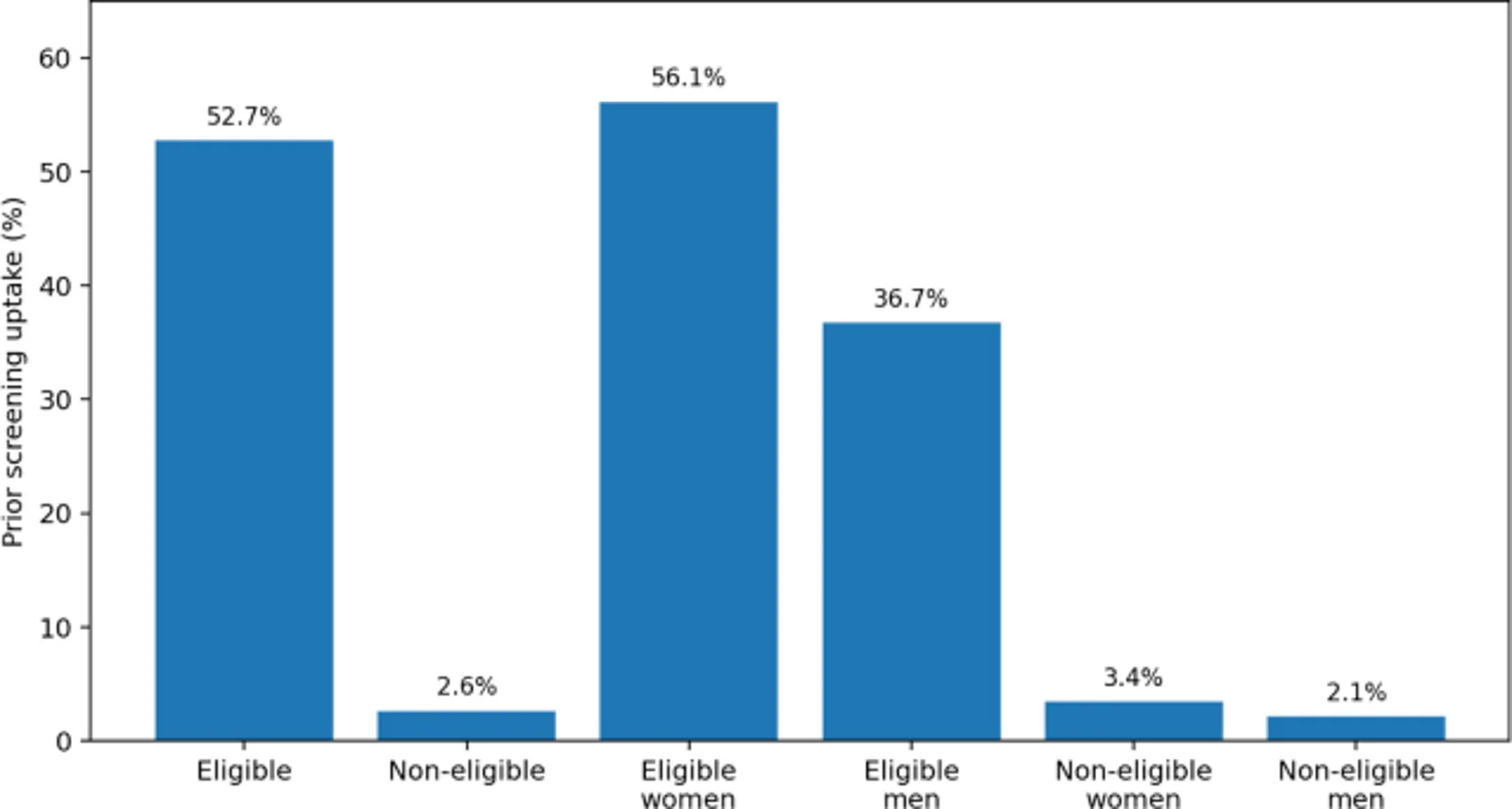
Prior screening uptake according to screening eligibility status. Note: Overall, prior screening uptake was 89/169 (52.7%) among eligible participants and 2/77 (2.6%) among non-eligible participants. In sex-specific descriptive strata, prior screening uptake was 78/139 (56.1%) among eligible women, 11/30 (36.7%) among eligible men, 1/29 (3.4%) among non-eligible women, and 1/48 (2.1%) among non-eligible men

In the eligibility-stratified analysis, 89/169 (52.7%) eligible participants had ever been screened compared with 2/77 (2.6%) non-eligible participants, χ²(1, *N* = 246) = 56.89, *p* < 0.001, Cramer’s V = 0.48. Sex-specific descriptive stratification showed that prior screening was reported by 78/139 (56.1%) eligible women, 11/30 (36.7%) eligible men, 1/29 (3.4%) non-eligible women, and 1/48 (2.1%) non-eligible men. Accordingly, sex differences were not interpreted as simple behavioral differences independent of screening eligibility structure and sample composition. These eligibility-stratified differences in prior screening uptake are illustrated in Fig. 1.

In contrast to awareness, crude prior screening uptake did not differ significantly by occupation (24/78 [30.8%] vs. 67/168 [39.9%]; χ²(1, *N* = 246) = 1.90, *p* = 0.168, Cramer’s V = 0.09).

Among those who had not previously undergone screening, the most frequently reported barriers were perceiving oneself as healthy, lack of time, and insufficient knowledge about cancer screening. These item-level results are presented in Table 3.

**Table 3:** Screening Uptake, Barriers, and Post-brochure Responses

Variable	*n* (%)
Prior screening uptake	
Ever screened	91/246 (37.0)
Never screened	155/246 (63.0)
Eligibility-stratified prior screening uptake	
Eligible participants who had ever been screened	89/169 (52.7)
Non-eligible participants who had ever been screened	2/77 (2.6)
Sex-specific descriptive stratification	
Eligible women who had ever been screened	78/139 (56.1)
Eligible men who had ever been screened	11/30 (36.7)
Non-eligible women who had ever been screened	1/29 (3.4)
Non-eligible men who had ever been screened	1/48 (2.1)
Barriers to screening^*^	
I considered myself healthy	87/188 (46.3)
Lack of time	36/188 (19.1)
Lack of knowledge	28/188 (14.9)
Post-brochure responses	
Thoughts changed after brochure	171/247 (69.2)
Would undergo screening after brochure	197/246 (80.1)
Paired pre/post willingness analysis (complete cases only)	
Yes before brochure	122/182 (67.0)
Yes after brochure	137/182 (75.3)

*Barrier items are presented as response counts among all recorded barrier responses (*N* = 188); percentages in this subsection therefore refer to total barrier responses rather than total participants. The eligibility-stratified comparison for prior screening uptake was statistically significant, χ²(1, *N* = 246) = 56.89, *p* < 0.001, Cramer's V = 0.48. The paired pre/post willingness comparison was analysed using McNemar's exact test (*p* = 0.004)

After the brochure intervention, willingness to undergo screening increased from 122/182 (67.0%) to 137/182 (75.3%) among participants with paired pre- and post-brochure responses (McNemar’s exact *p* = 0.004). In addition, 171/247 (69.2%) participants reported a positive change in their thoughts about cancer screening after the brochure. Because these responses were collected immediately after the intervention, they were interpreted as short-term changes in intention rather than confirmed behavioral outcomes. In exploratory subgroup analyses, no statistically significant heterogeneity was observed according to eligibility, sex, age group, education, or occupation (all *p* ≥ 0.105).

In a sensitivity analysis excluding the 12 participants with a personal history of cancer, the main findings were unchanged, suggesting that this subgroup did not materially drive the principal results.

In the exploratory multivariable binary logistic regression model for prior screening uptake, healthcare-worker status showed a borderline association (aOR = 2.65, 95% CI [0.96, 7.34], *p* = 0.061), while eligible screening status remained strongly associated with prior screening uptake. Full model estimates are presented in Table 4.

**Table 4:** Exploratory multiple binary logistic regression for prior screening uptake

Variable	Comparison	aOR (95% CI)	*p*
Eligible screening status	Eligible vs. not eligible	31.43 (7.07–139.73)	< 0.001
Healthcare-worker status	Healthcare worker vs. non-healthcare	2.65 (0.96–7.34)	0.061
Education	High school vs. University+	1.83 (0.65–5.11)	0.251
Education	Illiterate vs. University+	0.95 (0.22–4.12)	0.942
Education	Primary/Middle school vs. University+	1.61 (0.56–4.58)	0.373
Marital status	Married vs. single	3.41 (0.98–11.93)	0.054
Marital status	Widowed vs. single	4.36 (0.83–22.97)	0.083
Family history of cancer	Yes vs. no	1.26 (0.67–2.36)	0.469
Personal history of cancer	Yes vs. no	0.76 (0.20–2.92)	0.686

Dependent variable: prior screening uptake (yes/no). This was an exploratory multivariable binary logistic regression model based on complete-case analysis (*n* = 246). Reference categories were: not eligible, non-healthcare, University+, single, no family history of cancer, and no personal history of cancer

*aOR* adjusted odds ratio, *CI* confidence interval

## Discussion

In this cross-sectional study conducted in a family medicine outpatient setting, we identified a clear awareness–behavior gap in relation to cancer screening. Although a considerable proportion of participants reported at least some awareness of cancer screening tests, prior screening uptake remained substantially lower. This pattern is broadly consistent with observational evidence showing that awareness alone does not reliably translate into actual participation in organised screening programmes and that screening behavior is shaped by a combination of knowledge, perceived need, practical access barriers, and interaction with healthcare services [8, 9]. 

In our sample, screening uptake appeared higher among women and older participants; however, this pattern should be interpreted in the context of underlying differences in screening eligibility by sex and age, rather than as a direct behavioral difference. Similar sex- and age-related gradients in screening participation have been reported in European and middle-income country populations [10, 11]. 

Educational attainment showed a descriptive trend toward higher screening uptake, although this was not statistically significant in adjusted analyses. In line with existing literature, education is often linked to health literacy, perceived susceptibility, and the ability to navigate healthcare systems, which may influence preventive service use. Previous studies suggest that limited health literacy may be associated with lower cancer screening participation [12, 13]. 

Although participants with a family or close-contact history of cancer descriptively demonstrated higher screening uptake, this association was not statistically significant in adjusted analyses. This finding suggests that the relationship between family history and screening behavior could not be clearly established within this sample. It should also be considered that the analysis may have been underpowered due to the relatively limited sample size. Nevertheless, existing literature indicates that family history is associated with increased perceived cancer risk, which may influence screening-related decision-making [14, 15]. 

The most commonly reported barriers to screening participation in our study—perceiving oneself as healthy, lack of time, and insufficient knowledge—mirror barriers described in recent international literature. These barriers highlight the combined influence of cognitive, emotional, and practical factors on screening decisions in routine care [9, 16].

The brochure-based information provided during the outpatient visit was associated with a short-term increase in screening-related willingness. This finding is consistent with existing evidence suggesting that low-intensity educational interventions delivered at the point of care may influence screening-related intentions. However, this increase should be interpreted cautiously, as intention does not necessarily translate into completed screening behavior in the absence of supportive system-level mechanisms. Previous studies have emphasized that education alone is often insufficient to achieve sustained improvements in screening uptake [17–19].

Recent evidence suggests that mHealth-based strategies, including digital reminder systems, may improve participation in cancer screening programs. Digital interventions, particularly SMS-based approaches, have been associated with increased screening uptake and appear to be acceptable in organized screening settings [4].

The finding that information obtained from health professionals was a common source of screening knowledge in our study further underscores the central role of family physicians and primary care teams as trusted providers of preventive guidance. Integrating standardized brief education with referral prompts and reminder workflows within primary care may help reduce missed opportunities for screening [9, 18].

Recent research has demonstrated a significant association between health literacy, cancer-related knowledge, and screening behaviors, suggesting that improving health literacy may contribute to increased screening participation [13, 20].

Several limitations should be considered when interpreting these findings. The single-center design and outpatient-based sample may limit generalizability to broader community populations. In addition, screening history and attitudes were self-reported, which may introduce recall and social desirability bias. Finally, the post-brochure assessment captured short-term changes in intention rather than verified screening completion, precluding conclusions about sustained behavior change [17, 18].

Despite these limitations, this study provides practical insights into modifiable barriers and facilitators of cancer screening in primary care. By leveraging routine patient contact, family medicine services can play a pivotal role in bridging the gap between awareness and action through integrated educational and system-based strategies [3, 18, 19].

### Limitations

This study has several limitations. First, the cross-sectional design precludes causal inference. Second, the questionnaire was developed for the study and did not undergo formal psychometric validation. Third, self-reported responses may be subject to recall and social desirability bias. Fourth, post-brochure findings reflect immediate intention rather than confirmed screening behavior. Fifth, 31.6% of the sample consisted of healthcare workers, which may have influenced baseline awareness levels and may limit generalisability to the broader primary care population. Finally, personal cancer history was available only as a yes/no variable in the retained analysable dataset, and cancer type could not be classified reliably.

## Conclusions

In conclusion, awareness of cancer screening did not consistently translate into screening participation, even in the presence of free national screening programs. The increase in willingness observed after a brief brochure-based intervention reflects a short-term change in intention rather than confirmed behavioral change. Descriptive differences across sociodemographic groups should be interpreted cautiously, given the influence of screening eligibility structure. From a public health perspective, integrating brief education with structured referral and reminder systems in primary care may support screening uptake, although this was not directly assessed in the present study.

Future research should evaluate multicomponent interventions using longitudinal designs and verified screening outcomes. 

## Supplementary Information

Supplementary Material 1.

Supplementary Material 2.

## Data Availability

The datasets used and/or analysed during the current study are available from the corresponding author on reasonable request.

## Abbreviations

HPV: Human papillomavirus
PSA: Prostate-specific antigen
FOBT: Fecal occult blood test
SPSS: Statistical Package for the Social Sciences
