# Supplementary material for: Knowledge, attitudes, and behaviors regarding cancer screening among adults in primary care
Source: BMC Public Health. 2026 Jul 10;26:2807. doi: 10.1186/s12889-026-28493-9 (PMC13637412; doi:10.1186/s12889-026-28493-9)
Supplement: Supplementary file 1 [file 12889_2026_28493_MOESM1_ESM.docx]

**Questionnaire on Knowledge, Attitudes, and Behaviors Regarding Cancer Screening Among Adults**

**A. DEMOGRAPHIC INFORMATION**

1. Age: ………..

2. Sex:

Female ( )	Male ( )

3. Marital Status:

Married ( )	Single ( )	Widowed/Divorced ( )

4. Educational Status:

( ) Illiterate

( ) Primary school

( ) High school

( ) University / Master’s degree

5. Occupation:

( ) Healthcare professional (Physician, Nurse, Dentist, Pharmacist, Medical Technician, Caregiver)

( ) Non-healthcare professional

6. Area of Residence:

( ) Urban area	( ) Rural area

7. Do you have social security?

( ) Yes	( ) No

**B. HEALTH INFORMATION**

8. Do you have any chronic disease requiring regular medication?

( ) Yes	( ) No

9. Have you ever been diagnosed with cancer?

( ) Yes (Please specify): ……….. ( ) No

10. Is there a history of cancer in your family or close social environment?

( ) Yes	( ) No

11. If yes, which relative?

( ) Spouse

( ) First-degree relative (Mother, Father, Sibling, Child)

( ) Second-degree relative (Aunt, Uncle, Niece/Nephew, Grandparent)

( ) Distant relative / Acquaintance

**Type of cancer (if known):**

( ) Breast cancer

( ) Cervical cancer

( ) Colorectal cancer

( ) Other (Please specify): ………..

12. Do you have knowledge about cancer screening tests?

( ) Yes	( ) Partially	( ) No

**If your answer is “No,” please proceed to Question 15.**

13. How did you learn about cancer screening tests?

( ) Family physician

( ) Physician from another specialty

( ) Nurse or other healthcare professionals

( ) Social media (Television, Internet)

( ) Relatives / acquaintances

( ) Other (Please specify): ………..

14. Which cancer screening tests are you aware of? (You may select more than one)

( ) Breast cancer

( ) Cervical cancer

( ) Prostate cancer

( ) Colorectal cancer

( ) Lung cancer

15. Have you ever undergone cancer screening?

( ) Never

( ) At least once

**If you have never undergone screening, please proceed to Question 18.**

16. Which cancer screening tests have you undergone? (For those who have been screened)

( ) Mammography

( ) Pap smear / HPV DNA

( ) PSA

( ) Colonoscopy

( ) Fecal occult blood test

17. What was the main factor that motivated you to undergo cancer screening?

( ) Recommendation of a family physician

( ) Recommendation of a physician from another specialty

( ) Recommendation from relatives / acquaintances

( ) Family history of cancer

( ) Cancer history among acquaintances

( ) Social media

18. What is your reason for not undergoing cancer screening?

( ) Fear of receiving bad news

( ) Embarrassment related to healthcare staff or procedures

( ) Lack of time

( ) Lack of knowledge about cancer screening

( ) Belief that I am healthy

( ) Belief that screening would not be beneficial

( ) Lack of accessibility

( ) Other (Please specify): ………..

19. If you have not undergone screening, would you like to do so?

( ) Yes	( ) No

20. If yes, which screenings would you like to undergo?

( ) Breast cancer

( ) Cervical cancer

( ) Colorectal cancer

Participants will be informed about cancer screening tests.

**POST-INFORMATION**

21. After receiving information, has your opinion about undergoing cancer screening changed?

( ) Yes	( ) No

22. Would you undergo cancer screening?

( ) Yes	( ) No

23. If yes, which cancer screening test would you consider?

( ) Mammography

( ) Pap smear / HPV DNA

( ) Colonoscopy

( ) Fecal occult blood test

**END OF QUESTIONNAIRE. THANK YOU FOR YOUR PARTICIPATION.**
