# Supplementary material for: Knowledge, attitudes, and behaviors regarding cancer screening among adults in primary care
Source: BMC Public Health. 2026 Jul 10;26:2807. doi: 10.1186/s12889-026-28493-9 (PMC13637412; doi:10.1186/s12889-026-28493-9)
Supplement: Supplementary file 2 [file 12889_2026_28493_MOESM2_ESM.docx]

***CERVICAL CANCER***

*● It is the* ***third most common cancer among women worldwide****, after breast cancer.
● It can be detected* ***10–15 years in advance*** *through screening tests.
● When detected early, the* ***chance of successful treatment is 100%****.
● Women aged* ***21–65 years*** *should undergo a* ***Pap smear test every 3 years****, or women aged* ***30–65 years*** *should be screened every* ***5 years with a Pap smear test combined with an HPV-DNA test****.
● The screening test is a* ***simple and painless procedure*** *performed during a gynecological examination.*

*♥* ***Early detection saves lives in cancer.*** *♥* ***Have the appropriate screening test at a time suitable for you for early diagnosis.*** *♥* ***If you have not undergone screening, please apply to KETEM (Cancer Early Diagnosis, Screening and Training Center) or consult your family physician.*** *♥♥♥*

***COLORECTAL CANCER***

*● It is the* ***second most common cancer in women*** *and the* ***third most common cancer in men worldwide****.
● It may not cause symptoms until the cancer grows.
● It can be detected at an early stage through screening tests.
● In* ***men and women aged 50–70 years****, a* ***fecal occult blood test*** *should be performed* ***every 2 years****,
● In individuals aged* ***50–70 years****, a* ***colonoscopy should be performed every 10 years****.*

***BREAST CANCER***

*● It is the most common type of cancer among women worldwide.
● It can be detected at an early stage through physical examination and screening tests.
● When diagnosed at an early stage, the chance of recovery is* ***98%****.
● Performing* ***breast self-examination (BSE)*** *once a month,
● Undergoing a* ***clinical breast examination*** *once a year,
● Women aged* ***40–69 years*** *should have a* ***mammography every 2 years****.*
